# SEOM clinical guidelines in advanced and recurrent breast cancer (2018)

**DOI:** 10.1007/s12094-018-02010-w

**Published:** 2019-01-08

**Authors:** J. I. Chacón López-Muñiz, L. de la Cruz Merino, J. Gavilá Gregori, E. Martínez Dueñas, M. Oliveira, M. A. Seguí Palmer, I. Álvarez López, S. Antolin Novoa, M. Bellet Ezquerra, S. López-Tarruella Cobo

**Affiliations:** 1 Servicio de Oncología Médica, Hospital Virgen de la Salud, Avda. de Barber, 30, 45004 Toledo, Spain; 2 GEICAM, Madrid, Spain; 3 Servicio de Oncología Médica, Medicine Department, Hospital Virgen Macarena, University of Sevilla, Seville, Spain; 4 Servicio de Oncología Médica, Fundación Instituto Valenciano de Oncología, Valencia, Spain; 5 Servicio de Oncología Médica, Hospital Provincial de Castellón, Castellón, Spain; 6 Vall d’Hebron University Hospital and Vall d’Hebron Institute of Oncology, Barcelona, Spain; 7 Servicio de Oncología Médica, Corporació Sanitaria Parc Tauli, Sabadell, Barcelona Spain; 8 Servicio de Oncología Médica, Hospital Universitario Donostia/Donostiako Unibertsitate Ospitalea, Donostia, Spain; 9 Servicio de Oncología Médica, Hospital Universitario A Coruña, A Coruña, Spain; 10 Vall d’Hebron University Hospital and Vall d’Hebron Institute of Oncology (VHIO), Barcelona, Spain; 11 Servicio de Oncología Médica, Hospital General Universitario Gregorio Marañón, Instituto de Investigación Sanitaria Gregorio Marañón (IiSGM), Universidad Complutense, CiberOnc, GEICAM, Madrid, Spain

**Keywords:** Advanced, Loco-regional recurrence, Breast cancer, SEOM, Guidelines

## Abstract

Although the metastasic breast cancer is still an incurable disease, recent advances have increased significantly the time to progression and the overall survival. However, too much information has been produced in the last 2 years, so a well-based guideline is a valuable document in treatment decision making. The SEOM guidelines are intended to make evidence-based recommendations on how to manage patients with advanced and recurrent breast cancer to achieve the best patient outcomes based on a rational use of the currently available therapies. To assign a level of certainty and a grade of recommendation the United States Preventive Services Task Force guidelines methodology was selected as reference.

## Introduction

Breast cancer (BC) represents the first cause of invasive cancer in the Spanish women, accounting for 29% of all female cancers. According to recent data, 26,730 new cases and 6477 deaths are estimated annually in Spain [1].

Metastatic BC (MBC) remains virtually an incurable disease, with reported median overall survival (OS) of approximately 2 years. However, improved OS (up to 5 years) has been observed recently for certain subtypes, particularly in HER2-positive disease. De novo metastatic disease has better 5-year OS that recurrent MBC and prognosis appear to improve over time [2].

Patients with loco-regional recurrence (LRBC) may or may not be amenable to radical local treatment. Overall, according to a Spanish study, an increase in OS has been observed in the recent years in LRBC patients [3].

## Methodology

The SEOM guidelines have been developed with the consensus of ten breast cancer oncologists from the cooperative groups GEICAM (Spanish Breast Cancer Research Group) and SOLTI (Spanish Collaborative Group for the Study, treatment and other experimental strategies in solid tumors). To assign a level of certainty (LC) and a grade of recommendation (GR) to the different statements described in the clinical guidelines, the United States Preventive Services Task Force (USPSTF) guidelines methodology was selected as reference, as the previously adopted for the former version of SEOM recommendations [4] (Table 1).

**Table 1:** Strength of recommendation and level of certainty according to the United States Preventive Services Task Force (USPSTF) after July 2012 [4]

Category	Definition
Strength of recommendations (grade)	
A	The USPSTF recommends the service. There is high certainty that the net benefit is substantial
B	The USPSTF recommends the service. There is high certainty that the net benefit is moderate or there is moderate certainty that the net benefit is moderate to substantial
C	The USPSTF recommends selectively offering or providing this service to individual patients based on professional judgment and patient preferences. There is at least moderate certainty that the net benefit is small
D	The USPSTF recommends against the service. There is moderate or high certainty that the service has no net benefit or that the harms outweigh the benefits
I	The USPSTF concludes that the current evidence is insufficient to assess the balance of benefits and harms of the service. Evidence is lacking, of poor quality, or conflicting, and the balance of benefits and harms cannot be determined
Levels of certainty regarding net benefit	
High	The available evidence usually includes consistent results from well-designed, well-conducted studies in representative primary care populations. These studies assess the effects of the preventive service on health outcomes. This conclusion is therefore unlikely to be strongly affected by the results of future studies
Moderate	The available evidence is sufficient to determine the effects of the preventive service on health outcomes, but confidence in the estimate is constrained by such factors as the number, size, or quality of individual studies; inconsistency of findings across individual studies; limited generalizability of findings to routine primary care practice; lack of coherence in the chain of evidence. As more information becomes available, the magnitude or direction of the observed effect could change, and this change may be large enough to alter the conclusion
Low	The available evidence is insufficient to assess effects on health outcomes. Evidence is insufficient because of: the limited number or size of studies; important flaws in study design or methods.; inconsistency of findings across individual studies; gaps in the chain of evidence; findings not generalizable to routine primary care practice; lack of information on important health outcomes. More information may allow estimation of effects on health outcomes

## General overview of advanced breast cancer

### Goals of treatment

The two main goals for the treatment of MBC patients are to improve survival and to optimize the quality of life [5]. For LRBC patients for whom a radical approach is not feasible, the aims of the treatment are similar to that in MBC patients. Conversely, for those LRBC amenable to a local treatment the objectives will be to eradicate all macroscopic diseases and to improve both disease-free survival and overall survival.

- Since the diagnosis of advanced or recurrent BC is made, patients should also be offered appropriate multidisciplinary care, as it may have an impact in OS, including symptom-related intervention (*LC high; GR A*).
- Research is a priority in this setting. Participation in well-designed, independent, prospective trials should be offered to all eligible patients, whenever possible (*LC high; GR A*).

For all indications and breast cancer types, palliative treatment is strongly recommended when indicated.

### Diagnosis of recurrence and metastatic disease

- Clinical loco-regional recurrence should be confirmed by biopsy in all LRBC before planning any therapeutic strategy (*LC high; GR A*).
- Both in LRBC and in MBC (primary or relapsed) the histologic analyses should be performed if possible. In recurrences, it will serve to confirm neoplastic recurrence and to recheck histologic subtype, as changes in hormone receptors (HR) and HER2 between primary tumor and recurrences have been reported [6] (*LC high; GR A*).

### Staging

- For both MBC and LRBC clinical evaluation should include medical history and physical examination. Minimal staging workup should include imaging techniques of chest, abdomen and bone, hematology and biochemistry. The recommended imaging techniques are body TC and bone scintigraphy (*LC moderate; GR B*).
- The role of tumor markers (TM) in the follow-up of early BC patient is controversial [7] (*LC low; GR C*). Once recurrence is diagnosed, basal TM (CEA, CA15.3 and/or Ca 27.4) may be performed. Because, if elevated, may help to monitor disease response to therapy, especially in the presence of non-measurable disease (*LC low; GR C*). However, treatment decisions should not be based only on the variation of TM levels (*LC moderate; GR A*).
- Overall, the use of systematic brain image in all patients in the absence of suspicious symptoms is not recommended, even in HER2-positive disease (*LC moderate; GR B*).
- The use of Positron emission tomography (PET) is controversial in MBC, but can be used instead of TC and bone scan, if available (*level of evidence low, GR B*) [8]. In the postoperative surveillance  PET/TC is recommended only in cases of equivocal and conflicting findings [9] (*LC low; GR C*).

## Loco-regional recurrence management

### Local therapy

#### Local only recurrence

Patients with local and regional disease are divided into three groups.

- Initial treatment with lumpectomy + radiation therapy (RT): treat recurrence with total mastectomy + axillary lymph node staging if level II/III axillary dissection not previously done [10] (*LC moderate; GR B*). Limited data suggest that a repeated sentinel node biopsy may be successfully performed in patients who have previously undergone breast-conserving therapy and sentinel node biopsy [11] (*LC low; GR C*). For isolated ipsilateral breast cancer recurrences, breast conservative surgery plus partial breast irradiation is an alternative option [12] (*LC moderate; GR B*).
- Initial treatment with mastectomy and no prior RT: treat recurrence with surgical resection if possible + RT [13, 14] (*LC low; GR B*).
- Initial treatment with mastectomy + level I/II axillary dissection and prior RT: surgical resection if possible [13] (*LC low; GR B*). Limited data regarding additional irradiation [15] (*LC low; GR C*).

#### Regional only or local and regional recurrence

- Axillary recurrence: surgical resection if possible + RT if possible [16] (*LC moderate; GR B*).
- Supraclavicular and internal mammary node recurrence: RT if possible [17] (*LC moderate; GR B*).

Patients with disease not amenable to radical local treatment should be treated with induction chemotherapy (CT), endocrine or anti-HER2 therapy when indicated, and then palliative radiation, which is mandatory if the patient is radiation naïve [14, 18] (*LC low; GR B*).

### Systemic therapy

CT after first local or regional recurrence improves long-term outcomes primary in ER negative disease. Endocrine therapy in this setting improves long-term outcomes for ER positive disease [19] (*LC moderate; GR B*). In case of HER2-positive disease and the absence of previous anti-HER2 adjuvant treatment, trastuzumab is indicated [20] (*LC moderate; GR B*). However, the optimal strategy in case of previous adjuvant anti-HER2 treatment is unknown. CT is indicated for endocrine-resistant disease, as first line in triple-negative disease and combined with anti-HER2 drugs in HER2-positive disease.

In patients with disease not amenable to radical local treatment, the choice of palliative systemic therapy should be made according to the principles defined for metastatic BC (*LC high; GR A*).

## Endocrine therapy in advanced HR-positive/HER2-negative breast cancer

Since the last SEOM guideline for MBC on 2015 several advances have occurred in the treatment of endocrine therapy (ET) of luminal MBC [21]. Endocrine therapy includes ER-targeting drugs as single agents or in combination with drugs targeting pathways involved in hormone resistance such as the mTOR inhibitor, everolimus (PIK3/AKT/mTOR pathway) and CDK4/6 inhibitors (cell cycle pathway). ER-targeting drugs can act by lowering the levels of circulating estrogens or those acting directly in the ER (Table 2).

**Table 2:** Common classes of endocrine therapy

Mechanism of action	Class	Agent
Estrogen receptor blockage	SERM	Tamoxifen, toremifen
	SERD	Fulvestrant
Estrogen deprivation	Ovarian ablation	Surgery, radiation
	Ovarian suppression with GnRH analogs	Goserelin
		Triptorelin
		Leuprolide
	NSAI	Anastrozole
		Letrozole
	SAI	Exemestane
Unknown	Progestins	Megestrol acetate
		Medroxyprogesterone acetate
	High-dose estrogens	Diethylstilbestrol (DES)

Mechanism of action

*SERM* selective estrogen receptor modulator, *SERD* selective estrogen receptor downregulator (Fulvestrant 500 mg/month with loading dose is the recommended dosage), GnRH gonadotropin-hormone releasing-hormone, *NSAI* non-steroideal aromatase inhibitors (3rd generation), *SAI* steroidal aromatase inhibitors (3rd generation)

### General considerations

The preferred treatment for luminal ABC is ET in the majority of cases. Sequential ET should be used as long as the patient seems to be benefiting from ET and does not have evidence of immediately life-threatening disease or rapid progression of visceral disease with organ dysfunction (visceral crisis), or when there is an evidence of endocrine resistance. The presence of visceral involvement alone is not a contraindication for the use of ET (*LC high; GR A*).

The choice of which ET to use in each situation should take into consideration: (1) prior (neo) adjuvant ET vs “de novo” ABC; (2) Disease-free interval; (3) response to prior ET; (4) burden of disease and symptoms; (5) menopausal status; (6) comorbidities; (7) patient preferences and (8) costs and availability.

Since eventually all patients develop resistance or primarily resistant to ET, this must be considered before starting any therapy (first or next lines). Although a clear and consistent definition of resistance to ET is lacking, endocrine resistance has been defined by consensus (Table 3), as well as the type of ET that should be used in first and second lines (Table 4) [5, 22].

**Table 3:** Definition of endocrine resistance levels [5, 22]

Primary endocrine resistance	Relapse while on the first 2 years of adjuvant endocrine therapy (ET), or progressive disease (PD) within first 6 months of first-line ET for ABC, while on ET
Secondary endocrine resistance	Relapse while on adjuvant ET but after the first 2 years, or relapse within 12 months of completing adjuvant ET, or PD ≥ 6 months after initiating ET for ABC, while on ET

**Table 4:** Consensus definition of 1st and 2nd lines of endocrine therapy (ET) [5, 22]

1st line ET: (endocrine sensitive patients)	Newly diagnosed (de novo) ABC
	Relapse > 12 months from completion of (neo) adjuvant endocrine therapy with no treatment for advanced or metastatic disease (treatment naïve in the advanced setting)
2nd line ET	Relapse on or within 12 months from completion of (neo) adjuvant endocrine therapy with no treatment for advanced or metastatic disease (early relapse)
	Progression after 1st line of endocrine therapy for advanced disease (as described before)

The optimal sequence of endocrine-based therapy is uncertain. Available options for pre- and perimenopausal women with ovarian function suppression (OFS)/ovarian function ablation (OFA), and post-menopausal women include aromatase inhibitor (AI), tamoxifen, fulvestrant, AI/fulvestrant plus CDK 4/6 inhibitor, and ET plus everolimus. In later lines, also megestrol acetate and oestradiol, as well as repetition of previously used agents, may be used.

Besides the ER/PR positivity, as defined by international guidelines [23], we do not have another useful biomarker to select ET.

As the blockage of the estrogen signal is the mainstay of the treatment and the estrogen level varies depending on the menopausal status, it is important to define the situation of each patient (Table 5) [24].

**Table 5:** Definitions and menopausal status [24]

Menopause	Is the permanent cessation of menses
Reasonable criteria for determining menopause include any of the following	Prior bilateral oophorectomy
	Age ≥ 60 years
	Age < 60 years and amenorrheic for 12 or more months in the absence of chemotherapy, tamoxifen, toremifene, or ovarian suppression and follicle-stimulating hormone (FSH) and estradiol in the postmenopausal range
	If taking tamoxifen or toremifene, and age < 60 years, then FSH and plasma estradiol level must be in postmenopausal ranges
It is not possible to assign menopausal status to women who are receiving an LHRH agonist or antagonist	
In therapy-induced amenorrhea, oophorectomy or serial measurement of FSH and/or estradiol are needed to ensure postmenopausal status if the use of aromatase inhibitors is considered as a component of endocrine therapy	

### Postmenopausal women

#### First-line setting

- *Third generation AIs* Anastrozole, letrozole (non-steroidal aromatase inhibitors, NSAI) and exemestane (steroidal aromatase inhibitor, SAI) are superior to tamoxifen. There are no differences in efficacy between the three *AIs* [25] (*LC high; GR A*).  
- Fulvestrant 500 mg has better progression free survival (PFS) than anastrozol (18 vs 13 months), without differences in OS in patients without prior exposure to ET, particularly in those with non-visceral disease [26].
- The combination of an NSAI or fulvestrant plus CDK4/6 inhibitor has consistently shown to increase PFS in about 10 months compared to ET alone, although with more toxicity, and the effect is consistent in all trials and in all clinical subgroups.

The three options (AI, fulvestrant 500 mg and AI/fulvestrant + CDK4/6 inhibitor) are valid for the first line. Tamoxifen is also an option, if others cannot be used. The combination of ET plus CDK4/6 inhibitor is the preferred one if no other contraindications are present. [5, 22, 25, 26] (*LC high; GR A*).

- For patients that received chemotherapy as first line of treatment, maintenance ET is a reasonable option, although not assessed in clinical trials [5, 22]. There is somewhat less evidence for combination of ET with CDK4/6 inhibitors in this situation; so, our recommendation is to consider the use of ET alone, taking into account the toxicity and QoL variables (*LC low; GR B*).

#### Second-line setting

The second line will depend on which drug has been used as first line.

- AI is better than progestin, and similar to fulvestrant 250 mg. In second line fulvestrant 500 mg is better than 250 mg [21].
- For those patients treated with prior AIs, fulvestrant 500 would be the optimal option. [27]. Also, the alternate class of AI can be considered. Switching between steroidal and non-steroidal AIs produces modest additional clinical benefits, suggesting partial non-cross-resistance between the classes of inhibitor. However in these circumstances, the response rates to the second AI have generally been low [28].
- The combination of CDK4/6 inhibitors + fulvestrant has proven to be better than fulvestrant alone in patients without prior exposure to CDK4/6 inhibitors after progression to AIs, with increase in PFS [29–31] (*LC high; GR A*).
- Also, the combination of the mTOR (mammalian target of rapamycin) inhibitor everolimus with ET (exemestane in a phase III trial, and also with tamoxifen and fulvestrant in phase II trials) after progression to an AI leads to an increased PFS compared with ET alone, but with worse tolerance (see review in next section).

There are no data to define the best choice of treatment after progression to a CDK4/6 inhibitor in first line.

#### Subsequent lines of therapy

There is very limited information from prospective trials in patients with prior exposition to more than two lines of ET. In cases where a positive effect has been achieved with prior ET, those ET not previously used and progestins and other ET (Table 2) can be tested [5, 22] (*LC moderate; GR B*).

### Premenopausal women

- There are less data for premenopausal women treated with endocrine therapy alone, as many trials with ET have included mainly postmenopausal women. However, the old data with OFS in combination with tamoxifen, small trials with OFS and AI or fulvestrant and data of premenopausal patients included in new trials of ET with CDK4/6 inhibitors have proven that the benefit in premenopausal women is comparable to that obtained in postmenopausal [32, 33]. For all these reasons the international consensus is that the optimal management of pre/perimenopausal patients with luminal ABC consists of the induction of OFS or OFA in combination with another endocrine agent. Once the patient has been rendered postmenopausal, recommendations for postmenopausal apply (*LC high; GR A*).
- Adequate OFS for ABC premenopausal patients can be obtained through bilateral ovariectomy, continuous use of  LHRH agonists or OFA through pelvic RT (this latter is not always effective, and therefore is the least preferred option) [5, 22] (*LC high; GR A*).
- For those patients that do not desire ovarian suppression, tamoxifen is a reasonable option (*LC high; GR B*).

### Endocrine therapy in men

There are few data in these populations. International guidelines recommendations are that treatment should be chemical castration with GnRH analogs and then combination with ET as in postmenopausal women. Also, for those that do not want castration tamoxifen is a reasonable option [5] (*LC moderate; GR A*).

### Chemo-endocrine therapy

There is no clear evidence that concomitant use of ET plus chemotherapy results in improvement in OS. Therefore, this combination should be discouraged outside a clinical trial [34] (*LC low; GR D*).

### Duration of ET

ET should be continued until progressive disease or toxicity. For those patients treated with combined therapy (CDK4/6 inhibitors or everolimus) who have severe toxicity to the non-hormone component of the combination ET alone can be used until progression (*LC high; GR A*).

## Targeted therapy in advanced breast cancer

### CDK4/6 inhibitors: palbociclib, ribociclib and abemaciclib

The combination of a CDK 4/6 inhibitor with an AI is the preferred first-line option for most patients with endocrine-sensitive HR-positive/HER2-negative metastatic breast cancer (Table 2) [35–37] (*LC high; GR A*).

Recent evidence suggests the efficacy of the combination of CDK4/6 inhibitor with fulvestrant in first-line setting and endocrine-sensitive disease [30] (*LC high; GR A*).

The option of CDK4/6 inhibitor with an AI would also be appropriate for those patients who have not received prior treatment with a CDK4/6 inhibitor (*LC moderate; GR B*).

For patients with endocrine-resistant HR-positive/HER2-negative disease (Table 2) ABC, the combination of a CDK4/6 inhibitor with fulvestrant is the preferred option. [29–31, 38] (*LC high; GR A*).

Both combinations (CDK/AI and CDK/fulvestrant) are applicable regardless of the patient’s menopausal status, although pre/perimenopausal patients additionally will require ovarian function ablation or suppression.

Considering all these data, a CDK4/6 inhibitor should be added to endocrine therapy, at the latest when starting second-line endocrine therapy. Because the side effect profile of the CDK4/6 inhibitors appears substantially more tolerable than that seen with everolimus, the panel of experts recommends using CDK4/6 inhibitors rather than everolimus as the initial-targeted therapy to partner with ET.

With the exception of HR status, at present there are no validated predictive biomarkers to identify those women who could benefit from endocrine-based therapy with a CDK4/6 inhibitor (Table 6).

**Table 6:** Comparison of the status of authorization of CDK4/6 inhibitors

	EMA Indication
PALBOCICLIB	HR +/HER2 − ABC in combination with:
	An AI^a^
	Fulvestrant, in women previously treated with ET^a^
RIBOCICLIB	Women with HR +/HER2 − ABC, in combination with an AI or Fulvestrant as initial ET or in women who have received prior ET^a^
ABEMACICLIB	Women with HR +/HER2 − ABC in combination with an AI or Fulvestrant, as initial ET or in women previously treated with ET^a^

*HR *+*/HER2 *− *ABC* hormone receptor-positive and HER2-negative Advanced Breast Cancer, *AI* aromatase inhibitor, *ET* endocrine therapy

^a^Endocrine therapy must be combined with a luteinizing hormone–releasing hormone (LH–RH) agonist in pre or perimenopausal women

### mTOR inhibitors: everolimus

The combination of an AI with everolimus, an mTOR inhibitor, can be a valid treatment option for patients with endocrine-resistant HR-positive/HER2-negative metastatic breast cancer, since they are likely to obtain a significantly longer median PFS compared to aromatase inhibitor monotherapy (7.8 vs 3.2 months) [39, 40].

However, as an OS benefit could not be proved [41], when considering this targeted therapy, special attention must be paid to the increased toxicity including potentially severe side effects (e.g., non-infectious pneumonitis) (*LC high; GR B*). Primary prophylactic measures (e.g. mouthwashes with dexamethasone and meticulous oral hygiene) are recommended to prevent troublesome side effects [42]. Elderly patients should be closely monitored during treatment with proactive management of side effects.

With the exception of HR status, at present there are no validated predictive markers to identify those women who could benefit from endocrine-based therapy with a mTOR inhibitor.

### PARP inhibitors: olaparib and talazoparib

The poly-(ADP-ribose)-polymerase (PARP) inhibitors olaparib and talazoparib are both useful treatment options for patients with advanced germline *BRCA1/2*-mutated triple-negative breast cancer (TNBC) or HER2-negative luminal-like breast cancer [43, 44]. Specifically, olaparib has been recently approved by EMA to treat metastatic breast cancer after progression on chemotherapy. Patients should have received previous treatment in the form of (neo) adjuvant therapy or up to two lines of chemotherapy with anthracyclines and taxanes for metastatic disease, and must not have platinum-resistant disease.

This recommendation is based on longer PFS (approximately 3 months), higher overall response rate, good side effect profile and an improved quality of life with both PARP inhibitors compared to physician’s choice of standard chemotherapy (capecitabine, eribulin, or vinorelbine) in two randomized phase III trials (OlympiAD and EMBRACA trials) [43, 44] (*LC high; GR A*).

## Treatment of HER2-positive advanced breast cancer

### First-line therapy

The initial treatment approach to HER2-positive MBC must include a combination of chemotherapy and anti-HER2 therapy [45]. The same HER2 targeting agent should continue beyond progression through subsequent lines of treatment [46] (*LC high; GR A*).

Dual-blockade with trastuzumab, pertuzumab and taxanes is the treatment of choice in the first-line setting following the results of phase III CLEOPATRA trial that demonstrates a statistical significant improvement in PFS and OS from adding pertuzumab to trastuzumab and docetaxel [47] (*LC high; GR A*).

Replacing taxane with vinorelbine may be considered in certain circumstances [48] (*LC low; GR C*).

Of note, only 10% of CLEOPATRA patients were previously exposed to trastuzumab. In the PHEREXA trial testing trastuzumab plus capecitabine with or without pertuzumab, a smaller benefit from addition of pertuzumab to trastuzumab-exposed MBC patients in second-line setting was reported [49].

Trastuzumab emtansine (T-DM1) was non-inferior to trastuzumab-taxane in the phase III first-line MARIANNE study. However, there are no data regarding a head-to-head comparison between T-DM1 and dual HER2-blockade with trastuzumab, pertuzumab and a taxane. Consistently, T-DM1 is generally reserved for second-line setting [50]. One exception could be in cases of fast progression on/after adjuvant trastuzumab (6–12 months) or if the patient is not suitable for taxanes and dual blockade [51, 52] (*LC moderate; GR B*).

### Second-line therapy

T-DM1 is the recommended regimen for second-line therapy, as in the phase III EMILIA trial. It demonstrated superiority over lapatinib and capecitabine in terms of PSF and OS [53] (*LC high; GR A*). However, it should be noted that there are limited data about T-DM1 efficacy in pertuzumab exposed patients.

Pertuzumab, trastuzumab and chemotherapy may be considered as second line in patients previously not exposed to pertuzumab, and lapatinib and capecitabine are suitable options if T-DM1 was used as first line or it is contraindicated [51, 52] (LC moderate; GR B).

### Third-line therapy and beyond

Regimens currently recommended for first or second line should be considered for the later lines, if not used previously [5] (*LC low; GR C*).

T-DM1 demonstrated superiority over treatment of physician’s choice in third and later lines in phase III TH3RESA trial [54] (*LC high; GR A*).

Despite the proven activity of lapatinib and capecitabine in the second line [55], this combination was inferior to T-DM1 in EMILIA trial, so it preferably should be used afterwards (LC moderate; GR B).

Trastuzumab plus different chemotherapies (vinorelbine, capecitabine, gemcitabine) may be an option if not used previously (*LC low; GR C*).

Trastuzumab and lapatinib are also an option [56] (*LC moderate; GR B*).

The number and duration of each treatment line with HER2-targeted therapy and chemotherapy combinations cannot be established. The chemotherapy should continue for approximately 6 months (or longer) and/or to the time of maximal response, depending on toxicity and in the absence of progression. When chemotherapy is stopped, clinicians should continue the HER2-targeted therapy. Available data suggest that the benefit is maintained in third line and further therapy [51, 52] (*LC moderate; GR B*).

### HR-positive HER2-positive MBC

The first treatment approach in this population is HER2-targeted therapy plus chemotherapy (*LC high; GR A*).

In selected cases (including those with contraindications to chemotherapy, patient’s with a strong preference against chemotherapy or those with a long disease-free interval, minimal disease burden, in particular in terms of visceral involvement, and/or strong ER/PR expression) ET plus trastuzumab or lapatinib can be an option [57, 58] (*LC moderate; GR B*).

If a regimen of HER2-targeting therapy and chemotherapy is started, ET may be added to the HER2-targeted therapy when chemotherapy ends [51, 52] (*LC low; GR C*).

## Treatment of triple-negative advanced breast cancer

TNBC is characterized by the absence of expression of ER, PR, and HER2. TNBC is a heterogeneous entity. There is a significant overlap of TNBC with basal-like subtype by PAM50, although they are no synonyms [59].

Chemotherapy (CT) is the standard treatment for patients with TNBC [60]. The choice of the strategy and cytotoxic agents is conditioned by a large number of factors and must be considered individually. In general, sequencing single agent chemotherapy is preferred [61], limiting combination therapies for patients with aggressive, symptomatic or life-threatening disease [51] (*LC high; GR A*).

The optimal duration of CT is not well established, but generally a given regimen should be used until progression of disease or unacceptable toxicity [62] (as defined together with the patient) (*LC moderate; GR B*).

Given the aggressiveness of the disease and the limited effective treatment options, patients with metastatic TNBC should always be offered participation in well designed, prospective, independent trials whenever such trials are available, and the patient is willing to participate (*LC high; GR A*).

### First-line treatment

- In patients that are CT-naïve, anthracyclines or taxanes, either alone or in combinations are considered as first-line treatment [63] (*LC high; GR A*). This recommendation is also valid for patients with late recurrences (> 1 year) after completing (neo) adjuvant anthracyclines and/or taxanes.
- In patients with taxane-naïve and anthracycline-resistant MBC, or with anthracycline cumulative dose or toxicity who are being considered for further CT, taxane-based therapy, preferably as single agent would usually be considered as the therapy of choice (*LC high; GR A*). In patients pretreated with adjuvant taxanes and anthracyclines, other options such as vinorelbine [64, 65] and capecitabine [66] are also available (*LC moderate; GR B*).
- Bevacizumab, a humanized anti-VEGF monoclonal antibody, improves PFS and overall response rate (ORR), but not OS, when combined with taxanes or capecitabine in HER2-negative MBC patients [67–70] and may be considered for selected patients with aggressive or symptomatic disease (*LC moderate; GR C*). In the absence of predictive biomarkers, this benefit must be weighed against its toxicity profile (hypertension, proteinuria, and hemorrhagic events) [71]. Maintenance therapy with bevacizumab plus capecitabine (compared to bevacizumab alone) after induction first-line treatment with bevacizumab plus docetaxel improves PFS and OS for HER2-negative MBC patients (PFS 11.9 vs 4.3 months) [72]. Given these results, capecitabine plus bevacizumab may be considered for selected cases after initial CT with docetaxel plus bevacizumab (*LC low; GR C*).
- The combination of carboplatin and gemcitabine has been accepted as control arm by EMA and FDA in randomized trials, and actually showed a significant activity (PFS of around 5 months and median OS of around 1 year as first-line therapy) [73]. The combination is active in patients resistant to anthracyclines and taxanes, and it is an acceptable option in young patients with aggressive symptomatic disease (*LC moderate; GR B*).
- Carboplatin as first-line treatment for patients with TNBC and/or germline BRCA1- or BRCA2-associated MBC (gBRCA) was as effective as docetaxel in the randomized phase III TNT trial [74]. Although there were no differences in ORR or PFS in the unselected population (ORR, carboplatin 31.4% vs docetaxel 34.0%), gBRCA mutation carriers had an absolute difference of 34.7% in ORR (68% vs 33%, *p* = 0.03; biomarker, treatment interaction *p* = 0.01), which also translated in significant differences in PFS (6.8 vs. 3.1 months, *p* = 0.04). Based on these results, carboplatin can be considered as an option both for unselected TNBC patients (LC moderate; GR B) and for gBRCA MBC (LC high; GR A).
- Atezolizumab in combination with nab-paclitaxel has shown to improve PFS in patients with metastatic TNBC when compared to nab-paclitaxel alone [75]. This was achieved both in the intent-to-treat population (7.2 vs 5.5 months; HR 0.80) and among patients with PD-L1-expressing tumors in the infiltrating immune cells. In the intention-to-treat analysis, the median OS was 21.3 months with atezolizumab plus nab-paclitaxel and 17.6 months with placebo plus nab-paclitaxel (HR 0.84; *p* = 0.08), and among patients with PD-L1-positive tumors, the median OS was 25.0 months and 15.5 months, respectively. Although encouraging, additional results of trials testing immunotherapy agents plus chemotherapy must be awaited before a recommendation can be made to incorporate immunotherapy in first-line treatment for TNBC.

### Second and further lines of treatment

There is no limit to the number of therapy lines to be proposed to metastatic TNBC patients, as long as a good quality of life is maintained [51].

In patients pretreated with an anthracycline and a taxane, and who do not need combination CT, single-agent capecitabine, vinorelbine (oral or IV), and eribulin are the preferred choices [64, 65, 76, 77] (*LC moderate; GR B*). Additional choices include an alternative taxane (standard or nab-paclitaxel), rechallenge with anthracyclines (liposomal formulation), gemcitabine, or platinum agents [78] (*LC moderate; GR B*).

## Chemotherapy in luminal-advanced breast cancer

Chemotherapy should be the standard treatment for HR-positive MBC patients that are refractory to endocrine therapy, and for those women with triple-negative MBC [5] (see recommendations in the TNBC section).

Anthracyclines, taxanes, vinorelbine, capecitabine, gemcitabine and eribulin are reasonable and available options. The choice of the strategy and cytotoxic agents must be considered individually, taking into account previous therapies and their toxicities, tumor burden, time until recurrence after prior therapy, biological age, performance status, comorbidities, estimate life expectancy, need for a rapid disease/symptom control and patient’s preferences [5, 79]. No particular chemotherapeutic agent or regimen has been able to provide consistent gains in survival in these patients [79]. In general, sequencing single-agent chemotherapy is preferred [61, 80], limiting combination therapies for patients with aggressive, symptomatic or life-threatening disease (at least same efficacy, less toxicity) [5] (*LC high; GR A*).

Evaluation of response to chemotherapy should generally occur after two to four cycles depending on the dynamics of the disease, the location and extent of metastatic involvement and type of treatment. Imaging of target lesions may be sufficient in many patients (*LC moderate; GR B*) [5]. Subjective and objective toxicities must be evaluated repeatedly, as well as the symptoms and performance status. Duration of each regimen and the number of regimens should be tailored to each individual patient.

### First-line treatment

- In the absence of medical contraindications or patient concerns, anthracyclines or taxanes, either alone (preferably) or in form of combinations, are considered the first-line chemotherapy of choice, particularly in patients without prior adjuvant chemotherapy or with late relapses (*LC high; GR A*). After pretreatment with anthracyclines, the first options are taxanes, preferably using weekly paclitaxel or docetaxel every 3 weeks (*LC high; GR A*). Nanoparticle albumin-bound (nab)-paclitaxel as weekly or 3-weekly schedule is an alternative option (*LC moderate; GR B*).
- The combination of bevacizumab plus taxanes improves PFS and ORR, but not OS versus chemotherapy alone and should also be considered a first-line chemotherapy option in selected cases [69, 70, 81] (*LC moderate; GR B*) (see recommendations in the TNBC section).
- In patients pretreated with adjuvant taxanes and anthracyclines, other options such as vinorelbine [64, 65] and capecitabine [66] are also appropriate first-line chemotherapy options (*LC moderate; GR B*) (see recommendations in the TNBC section).

### Second and further lines of chemotherapy

- A large number of agents have shown activity as second-line chemotherapy and beyond in HR-positive MBC and may be suitable for sequential treatment in selected patients. Among them, capecitabine, vinorelbine, gemcitabine, nab-paclitaxel, liposomal doxorubicin and eribulin are approved options and can be appropriate therapies [65, 76, 77, 82, 83].
- In a phase III trial in patients pretreated with taxanes and anthracyclines, eribulin was not superior to the current standard capecitabine in PFS or OS. In another phase III trial, eribulin has shown a modest improvement in OS in patients with prior taxanes, anthracyclines and capecitabine. Therefore, it is the CT drug of choice in this population (*LC high; GR A*).

Considering these data, capecitabine is the most recommendable first option for HR-positive MBC patients pretreated with taxanes and anthracyclines, while eribulin can be administered after progression on capecitabine (*LC high; GR A*).

Drug rechallenge may be appropriate in selected cases; taxane rechallenge should not be performed within 12 months after the last taxane treatment [84] (*LC moderate; GR B*). Liposomal doxorubicin might be used in the same indications [85] (*LC low; GR C*).

## Special situations: CNS, bone, primary tumor

### Treatment of central nervous system metastases

Brain metastases is a frequent and challenging situation that affects up to 10–30% of all breast cancer patients during the course of their disease [86], being the HER2-positive and TNBC groups those with the highest incidence (up to 40%). Brain metastases are associated with the shortest survival time compared with other sites of metastatic disease in breast cancer [87], although a sizeable fraction of these patients may reach a prolonged survival (2 years and beyond), due to the higher efficacy of the novel local and systemic therapies.

A modified breast-graded prognostic assessment (GPA) index has recently been postulated [88, 89]. It integrates four simple clinical characteristics (age, Karnofsky score, number of brain metastases (BM) and breast cancer subtype) and may serve to guide further treatments in BM breast cancer patients.

#### Local therapies

*Surgery* Might be considered in specific circumstances: high breast-GPA index, 1–3 brain metastases, systemic disease under control, when BM are symptomatic and not responding to other therapies, and finally for diagnostic purposes [90] (*LC moderate; GR B*).

*Stereotactic radiotherapy* Stereotactic uses high conformal radiation that applies high doses of RT (15–25 Gy) in one or a few sessions [91]. This treatment is generally indicated in selected cases of oligometastatic disease (≤ 3 metastases) and can be considered an alternative to surgery [92] (*LC moderate; GR B*).

*Whole brain radiation therapy* (*WBRT*) Consists in the administration of 30 Gy in 10 fractions to the brain. This strategy, although palliative and indicated for symptomatic relief, has also an impact on BM breast cancer evolution as it has demonstrated a prolongation in OS versus best supportive care [93]. WBRT is generally recommended when there are multiple lesions (≥ 3 metastases), and/or when lesions are higher than 3 cm or have a volume of ≥ 25 cm^3^ [94] (*LC moderate; GR B*).

#### Systemic treatments

The value of systemic treatments on local control of brain metastases is unclear. A personalized approach attending BC subtype, disposable therapies and Karnofsky score is highly advisable (*LC moderate; GR B*).

*Chemotherapy* Although there are currently no specifically systemic treatments approved for BMBC, agents like capecitabine and topotecan, have demonstrated activity in this setting as with BM overall responses in the range of 38–60% [95–98]. In the case of combinations, cisplatin and etoposide, cisplatin and temozolomide, CMF and CAF schedules reach a response rate quite similar to those obtained in extracranial disease, ranging from 38 to 76% [95].

*Targeted therapies and endocrine*-*therapy* Nowadays, targeted therapies for BMBC remain confined to HER2 advanced BC disease, although evidence is not clear. Improved survival with trastuzumab in HER2-positive BM breast cancer has been attributed to a better control of extracranial systemic disease [99].

Lapatinib alone does not seem particularly active in BM breast cancer, although results seem to improve when combined with capecitabine [100].

With respect to T-DM1 a subset analysis of the EMILIA trial showed that among patients with treated and stable brain metastases at baseline (*n* = 95), a significant improvement in OS was observed in the T-DM1 arm with a hazard ratio of 0.38 (*p* = 0.008), median OS 26.8 vs 12.9 months [101]. More data from prospective trials are needed to ascertain the real efficacy of T-DM1 in BM breast cancer.

The anti-VEGF monoclonal antibody bevacizumab seems to improve results when combined with carboplatin [102, 103], but more solid evidences are needed.

Solid evidence for efficacy of endocrine therapy, PARP inhibitors, CDK4/6 inhibitors, in BM breast cancer is lacking.

### Role of local therapies in MBC

They are indicated for symptoms palliation and prevention of cancer-related complications (*LC moderate; GR B*). Evidence regarding survival prolongation is contradictory and elusive.

- *Local therapy of the primary tumor in the novo MBC* Routine breast surgery cannot be recommended in de novometastatic BC in patients who are asymptomatic at the site of their primary (*LC moderate; GR B*), provided that conflicting results from meta-analysis [104–106] and two prospective trials are obtained [107, 108].

#### The role of surgery for extracranial metastasis in MBC

- Can be considered after a balanced decision process and on a case-by-case basis (*LC low; GR C*).
- Oligometastatic disease in fit patients with long-term disease-free intervals or good response to previous systemic treatments could be considered as criteria for surgery with a curative intent (*LC low; GR C*).

#### The role of radiotherapy for extracranial MBC

- RT in MBC plays an important role in palliation (i.e., uncontrolled pain, spinal cord compression or fractures) (*LC high; GR A*).

### Treatment of bone metastases

Bisphosphonates and other osteoclast inhibitors have shown to be effective in reducing morbidity of metastatic bone disease with respect to skeletal-related events (SREs) and should be added to anti-tumor therapy in patients with bone metastases [109] (*Level of certainly high; GR A*).

- The most appropriate duration of anti-resorptive therapy (bisphosphonates, denosumab) in BC patients with bone metastases is unknown. Two randomized controlled trials have demonstrated that, after at least 1 year of standard monthly antiresorptive therapy, zoledronic acid every 12 weeks is non-inferior to every 4 weeks with respect to SREs [110, 111], and thus it may represent a new option in clinical practice (*LC high; GR A*).
